# Evaluation of the Larvicidal Efficacy of Five Indigenous Weeds against an Indian Strain of Dengue Vector,* Aedes aegypti* L. (Diptera: Culicidae)

**DOI:** 10.1155/2016/2857089

**Published:** 2016-01-31

**Authors:** Aarti Sharma, Sarita Kumar, Pushplata Tripathi

**Affiliations:** ^1^School of Life Sciences, Indira Gandhi National Open University, Maidan Garhi, New Delhi 110068, India; ^2^Department of Zoology, Acharya Narendra Dev College, University of Delhi, Kalka Ji, New Delhi 110019, India

## Abstract

*Background and Objectives*.* Aedes aegypti*, dengue fever mosquito, is primarily associated with the transmission of dengue and chikungunya in tropical and subtropical regions of the world. The present investigations were carried out to assess the larvicidal efficiency of five indigenous weeds against* Ae. aegypti. Methods. *The 1,000 ppm hexane and ethanol extracts prepared from the leaves and stem of five plants (*Achyranthes aspera*,* Cassia occidentalis*,* Catharanthus roseus*,* Lantana camara*, and* Xanthium strumarium*) were screened for their larvicidal activity against early fourth instars of dengue vector. The extracts which could cause 80–100% mortality were further investigated for their efficacy.* Results. *The preliminary screening established the efficacy of hexane extracts as compared to the ethanol extracts. Further investigations revealed the highest larvicidal potential of* A. aspera *extracts exhibiting LC_50_ value of 82.555 ppm and 68.133 ppm, respectively. Further, their leaf extracts showed 5–85.9% higher larvicidal activity and stem extracts exhibited 0.23- to 0.85-fold more efficiency than the other four extracts.* Conclusion. *The present investigations suggest the possible use of* A. aspera *as an ideal ecofriendly, larvicidal agent for the control of dengue vector,* Ae. aegypti*. Future studies are, however, required to explore and identify the bioactive component involved and its mode of action.

## 1. Introduction

Mosquitoes have attracted considerable attention worldwide being the most prevalent vectors of several lethal diseases, malaria, filariasis, chikungunya, yellow fever, dengue, and encephalitis, accounting for enormous mortality and morbidity. Since last few years, dengue fever has become the major public-health concern in tropical and subtropical regions of the world. It is considered the most rapidly spreading mosquito-borne disease with 30-fold rise in global occurrence since the past 5 decades. The incidence of dengue infections estimated by World Health Organization is about 390 million annually of which 96 million are supposed to be manifested clinically [1]. As per WHO reports, approximately 3900 million individuals, inhabiting over 128 endemic countries, are likely to be at the risk of dengue. In India, official records of the Union Health Ministry reveal a massive increase in dengue infections every year [2].

Till date, specific medications and vaccinations are not available commercially for treating dengue fever. The only approach followed to reduce the incidence of dengue is by the control of its vector,* Aedes aegypti *L., which is also the primary carrier of chikungunya virus and yellow fever virus. In the past, the control measures for mosquito vectors were based on the frequent and indiscriminate use of synthetic chemical-based insecticides, such as organochlorines, carbamates, organophosphates, and pyrethroids [3]. Nevertheless, the blind use of insecticides has resulted in the increased selection pressure on the mosquitoes leading to the development of insecticide resistance in them [4, 5]. Varying amount of resistance to commonly used insecticides, temephos, fenthion, malathion, and dichlorodiphenyltrichloroethane (DDT), has been reported by Tikar et al. [6]. In addition, it has raised many other concerns including toxicity to human beings, harm to nontarget population, long persistence in environment, and entry in the food chain [7].

Keeping in view the increasing documentation of negative environmental and health impact of synthetic insecticides and increasingly rigorous environmental directives about use of pesticides, the researchers have transformed their interest towards the development and use of botanical pest management products for controlling mosquitoes and other insects [8]. Botanicals are considered safe alternative to synthetic pesticides since they are biodegradable and safe for environment causing low toxicity to humans and nontarget organisms [9]. More than 2,000 plants species have already been known to possess chemical factors and metabolites of significance in pest control programs whilst products of approximate 344 species have been reported to encompass diverse activities against mosquitoes [10, 11]. A number of such plant products have been used for insect control since primordial time. Biologically active plant extracts have been well recognized for formulating an ecologically sound and environmentally accepted mosquito control program; more studies are being carried out to identify variety of bioeffective substances found in different plant species [8]. Several reports have established the efficacy of plant extracts and essential oils as efficient mosquito larvicides and repellents, without posing hazards of toxicity to humans [12–15]. Nevertheless, the mosquito control potential of weed plants has not been attempted at large scale leading to the availability of limited reports concerning the usefulness and promising use of certain weeds against* Ae. aegypti* as larvicidal, ovicidal, and oviposition deterrent agents [5, 16–18].

Weeds are nowadays preferable plants of research because of their easy availability, cultivability, undesirability, and need of management. Moreover, the beneficial utilization of weeds as mosquito control agents can boost the weed management programs. The present study assesses the mosquito control potential of five common indigenous weeds,* Achyranthes aspera, Cassia occidentalis, Catharanthus roseus, Lantana camara*, and* Xanthium strumarium*.* Achyranthes aspera* (Amaranthaceae), commonly known as prickly chaff flower, is an important medicinal herb found commonly on the roadsides throughout India. The extracts prepared from the leaves, flowers, and the seeds of* A. aspera* have been reported to possess biological activity against the fourth instars of* Anopheles subpictus* and* Culex tritaeniorhynchus* [19]. Certain bioactive components derived from this plant have been found to exhibit larvicidal, pupicidal, and growth regulatory activity against insects [20].* Cassia occidentalis* (Fabaceae) is an erect, lightly branched leguminous tree/shrub, commonly known as Coffee Senna or Coffee Weed, which is found in tropical, subtropical, and semiarid regions. In India it is widely prevalent as an opportunist that grows abundantly along roadsides immediately after rains [21]. The leaf extracts of* C. occidentalis* have been found to have effective activity against* Anopheles* sp. and* Cx. quinquefasciatus *larvae [22, 23]. Likewise,* Catharanthus roseus* (L.) (fam., Apocynaceae; formerly* Vinca rosea* L.), naturalised in all tropical countries and invasive in parts of Kenya, is reported to exhibit excellent control potential against* Ae. aegypti, An. stephensi*, and* Cx. quinquefasciatus* [9, 24]. Another weed selected for the current study,* Lantana camara* (Verbenaceae), commonly known as Wild Sage, an aggressive, obligate breeder weed that has invaded vast areas in many tropical and subtropical regions, has been regarded as one of the 10 most noxious weeds in the world [25].* L. camara* has also been reported to possess mosquito larvicidal and adulticidal activity against* Ae. aegypti, Cx. quinquefasciatus, An. culicifacies, An. fluviatilis*, and* An. stephensi* [26]. On the other hand,* Xanthium strumarium* (fam., Asteraceae; chotadhatura), which grows in tropical environments and is particularly abundant in India, Pakistan, and Sri Lanka, offers larvicidal and repellent potential against* An. culicifacies* Giles species A,* An. Stephensi, Cx. quinquefasciatus*, and* Ae. aegypti* [27].

Keeping in view above properties and possible mosquito control potential of these weeds, the leaves and stems of all these five weeds were assessed for their larvicidal potential against an Indian strain of dengue vector,* Ae. aegypti*. The investigations were carried out with an objective that the result of the study could be useful in promoting research aiming at the development of new eco-friendly alternative for mosquito control based on the biologically active plant sources.

## 2. Materials and Methods

### 2.1. Rearing of Mosquitoes

The present investigations employed the larvae of dengue fever mosquito,* Ae. aegypti*, which were collected from fields of Delhi and surrounding areas. Rearing of the mosquito vector was carried out in a laboratory maintained at 28 ± 1°C, 80 ± 5% RH and 14 : 10 L/D photoperiod as per the protocol described by Kumar et al. [28].

### 2.2. Collection of Plant Material

Five plant species of weeds were identified for the present investigations keeping in mind collecting species which were neither threatened nor endangered nor endemic. Moreover, selection of the plants was based on the literature survey, their easy availability, and uncomplicated cultivation being competent and invasive in nature. The five species of plants selected,* A. aspera, C. occidentalis, C. roseus, L. camara*, and* X. strumarium*, belonged to different families and also carried some medicinal importance.

**Figure 1:**
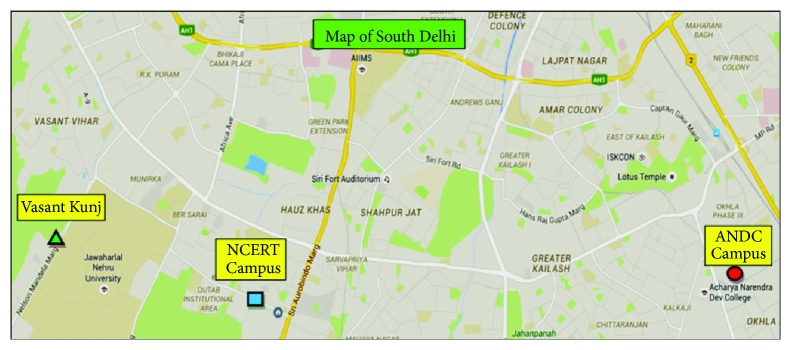
Different sites of the collection of different plant species in South Delhi, India. ● ANDC Campus [Latitude, Longitude (28.538548, 77.262919) 28°32′18.7728′′N and 77°15′46.5084′′E] (*Catharanthus roseus*). ■ NCERT Campus [Latitude, Longitude (28.538037, 77.192734) 28°32′ 16.9332′′N and 77°11′33.8424′′E] (*Lantana camara*). ▲ Vasant Kunj [Latitude, Longitude (28.547015, 77.161265) 28°32′49.2540′′N and 77°9′40.5540′′E] (*Achyranthes aspera, Cassia occidentalis*, and* Xanthium strumarium*).

The plants were collected from different sites of South Delhi, India (Figure 1), and brought to the laboratory in sterile polythene bags. The leaves and stems of each plant were separated and thoroughly washed with distilled water to clean dust or any other undesirable particles stuck to them. The plant parts were also carefully scrutinized for any disease or infection. The selected healthy parts were shade-dried at room temperature (27 ± 2°C) for approximate three weeks till they were dried completely. The five plant species and the parts used in the present investigations are summarized in Table 1.

### 2.3. Preparation of Plant Extracts

Plant extracts were prepared by maceration method in which the dried plant parts were ground with electric blender and then sieved to get fine powder. The 50 g of each dried and powdered part was weighed and soaked in 200 mL of ethanol and hexane solvent, separately in a stoppered conical flask (500 mL) for a definite period of time of 5–7 days with frequent agitation until soluble matter was dissolved. The extracts, thus obtained, were concentrated using a vacuum evaporator (Buchi Type) under low pressure keeping the temperature not more than their respective boiling point, that is, 78°C and 60°C. The concentrated extracts were stored in a refrigerator at 4°C as the stock solution of 1000 ppm prepared using ethanol as the solvent.

### 2.4. Screening of Extracts for Larvicidal Efficacy against* Ae. aegypti*

The larvicidal efficacy of all the extracts was investigated in two phases. In the first phase, the toxicity potential of 1000 ppm of each extract was screened against early fourth instars of* Ae. aegypti*. For screening, the active early fourth instars of* Ae. aegypti* were separated in batches of 20. Each batch was transferred in separated bowls containing 99 mL of distilled water which were then transferred to glass jars containing 100 mL of distilled water and 1 mL of an extract at 1000 ppm. Each bioassay had three replicates carried out concurrently at comparable conditions. Controls were exposed to the solvent, that is, ethanol alone.

The dead and moribund larvae were recorded after an exposure period of 24 h. The larvae were touched gently with the help of a glass rod and were considered dead in the absence of any signs of movement. The larvae were considered, however, moribund if they only moved a little without showing any kind of swimming or vigorous movement. The moribund larvae could never revive and thus were counted as dead larvae. The extracts that failed to give 80%–100% mortality at 1000 ppm after larval exposure for 24 h were rejected while extracts that resulted in mortality more than 80% after 24 h treatment were selected for further investigations.

### 2.5. Larvicidal Bioassay with Selected Plant Extracts against* Ae. aegypti*

In the second phase, the early fourth instars of* Ae. aegypti* were assayed with the selected extracts to assess the larvicidal efficacy of each extract in order to ascertain their probable use in the fields. The bioassays were performed at 28 ± 1°C in accordance with the procedure described by World Health Organization with slight modifications [29]. The graded series of each of the selected extracts was prepared using ethanol as the solvent. The bioassays were carried out at 20, 40, 60, 80, 100, 200, 400, 600, 800, and 1000 ppm. Three replicates were carried out at once for each concentration of each extract. Controls were exposed to the solvent, that is, ethanol alone.

### 2.6. Data Analysis

The tests which caused more than 20% larval mortality in controls and resulted in more than 20% pupae formation in any bioassay were discarded and conducted again. In case the larval mortality in control bioassays ranged between 5% and 20%, the control mortality was corrected using formula proposed by Abbott [30].

The obtained data was analysed by regression analysis using computerized statistical program SPSS (Version 22.0). The LC_50_ and LC_90_ values with 95% fiducial limits and chi-square were calculated in each bioassay for the assessment of significance and measurement of difference between the test samples.

## 3. Results

The screening for larvicidal potential of hexane and ethanol extracts prepared from the leaves and stems of five plant species,* A. aspera, C. occidentalis, C. roseus L. camara*, and* X. strumarium*, was carried out against early fourth instars of* Ae. aegypti. *The results obtained are presented in Table 1 which showed that 24 h exposure of the larvae with 1000 ppm hexane extracts could result in 100% mortality, irrespective of the parts from which they were prepared. On the other hand, when the larvae of* Ae. aegypti* were exposed to the leaf and stem extracts prepared in ethanol, the larval mortality ranged between only 5.0% and 50.0% (Table 1). The least larval mortality of 5.0% was caused by the exposure to stem ethanol extract of* L. camara *and that of* C. roseus*, whereas leaf ethanol extract of* C. roseus *resulted in highest observed mortality of 50.0%. Thus, all the ethanol extracts which could not result in significant larval mortality even at 1000 ppm were rejected for further investigations.

When the larvicidal potentials of the leaf and stem hexane extracts of five plant species were evaluated against early fourth instars of* Ae. aegypti*, all the extracts revealed their potency to inflict mortality in early fourth instars of* Ae. aegypti *(Tables 2 and 3). The data revealed that the larval exposure did not result in any pupal emergence indicating the selection of early instar stage. Moreover, the control or untreated group also did not exhibit any larval mortality or pupal emergence within 24 h.

**Figure 2:**
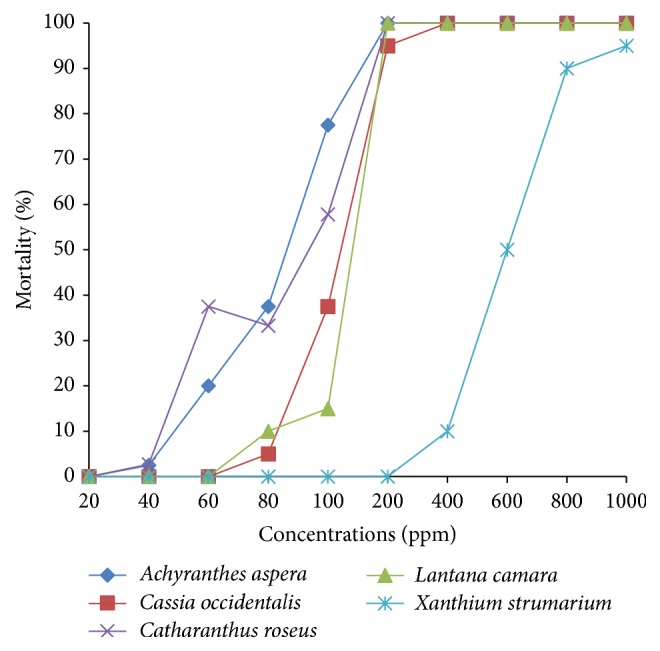
Percent mortality of hexane leaf extracts of different selected plants at different concentrations against early IV instars of* Ae. aegypti*.

**Figure 3:**
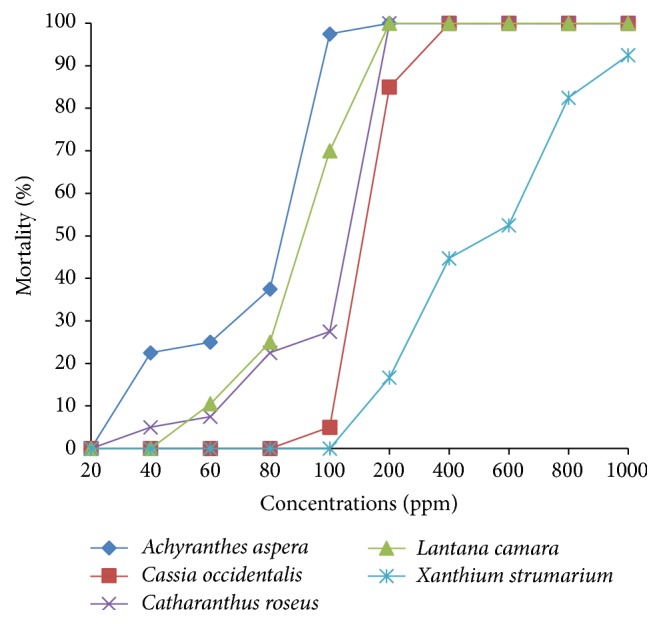
Percent mortality of hexane stem extracts of different selected plants at different concentrations against early IV instars of* Ae. aegypti.*

**Figure 4:**
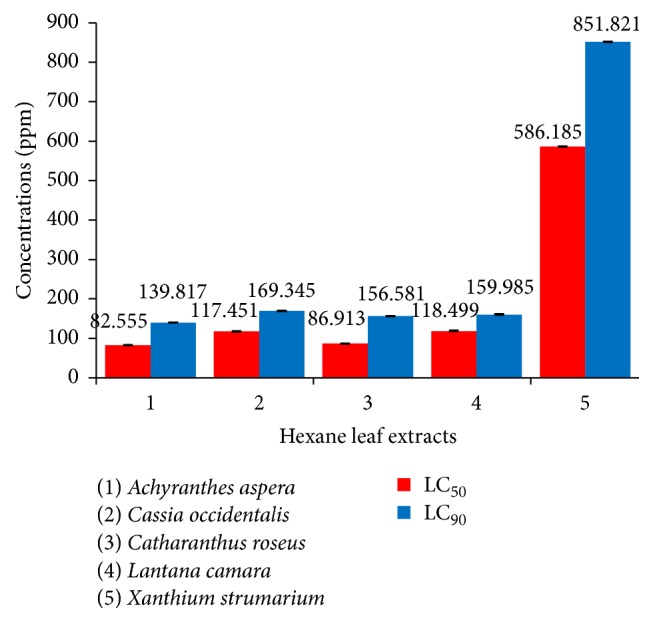
Comparative larvicidal activity of hexane leaf extract of different selected plants against early IV instars of* Ae. aegypti*.

**Figure 5:**
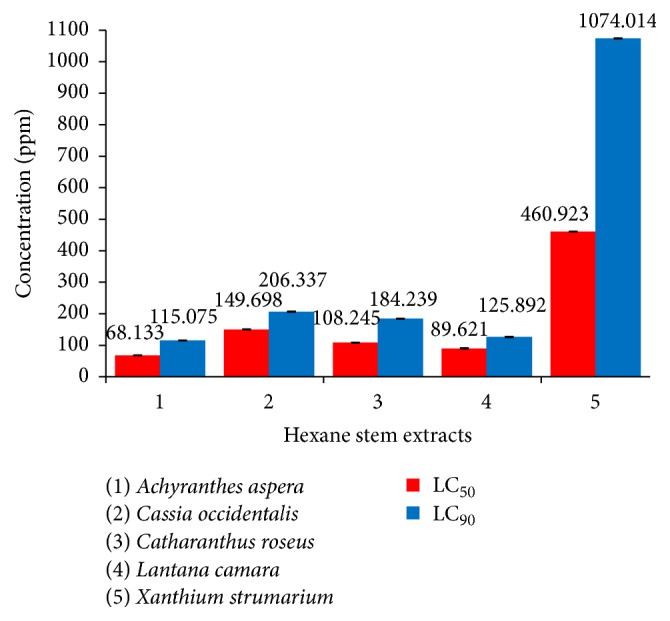
Comparative larvicidal activity of hexane stem extracts of different selected plants against early IV instars of* Ae. aegypti*.

Our investigations clearly revealed the highest larvicidal potential of* A. aspera *extracts against* Ae. aegypti *early fourth instars, whether prepared from leaves or stems, exhibiting LC_50_ value of 82.555 ppm and 68.133 ppm, respectively. The hexane leaf extract of* A. aspera *showed 5% to 85.9% higher larvicidal activity in comparison to that shown by remaining hexane leaf extracts (Figure 4). On the other hand, when the larvae were exposed to the hexane extracts prepared from the stems, the* A. aspera *extract proved to be 0.23- to 0.85-fold more efficient than the other four extracts (Figure 5). It is also significant to note the appreciable larvicidal efficacy of leaf hexane extract of* C. roseus *and stem hexane extract of* L. camara *revealing LC_50_ values of 86.913 ppm and 89.621 ppm, respectively. Nevertheless, stem hexane extract of* C. roseus *and leaf hexane extract of* L. camara *could also result in noticeable larval mortality, the sudden rise in mortality noticed on exposure to 100 ppm extracts (Figures 2 and 3).

On the other hand, 24 h exposure of the early fourth instars of* Ae. aegypti *to the leaf and stem hexane extracts of* X. strumarium *resulted in moderate toxic effects, the respective LC_50_ values obtained being 586.185 ppm and 460.923 ppm (Tables 2 and 3; Figures 4 and 5). The results showed that exposure to 100 ppm of leaf and stem hexane extracts of* X. strumarium* could not cause any larval mortality (Figures 2 and 3) whereas, on increasing the exposure concentration to 200 ppm, the hexane leaf extract was found to be ineffective, while the hexane stem extract could result in only 17% larval mortality as compared to 100% mortality obtained on exposure to* A. aspera *extracts.

On the contrary, when the early fourth instars of* Ae. aegypti *were exposed to* C. occidentalis *extracts, the hexane leaf extract showed higher efficacy as compared to the hexane stem extract, the respective LC_50_ values being 117.451 ppm and 149.698 ppm (Tables 2 and 3; Figures 4 and 5). It is remarkable to note that both the leaf hexane extract and leaf stem extract did not cause any appreciable mortality at lower concentrations after which a steep rise in larval mortality was noticed (Figures 2 and 3).

## 4. Discussion

The extensive use of synthetic chemical insecticides results in environmental degradation, hazards, and resistance in major vector species and this has necessitated leading the way towards the development of a more potent and environmentally friendly insecticide. Nowadays, the control of mosquitoes at larval stage is focused on the use of plant extracts. Plants produce various chemicals, many of which have medicinal, insecticidal, repellent, and growth regulatory properties [11].

The present investigations clearly revealed that when 1000 ppm hexane and ethanol stem and leaf extracts were screened for their larvicidal efficacy against early IV instars of* Ae. aegypti*, the hexane extracts exhibited significant larvicidal efficacy causing 100% larval mortality. Similar results were reported by Kumar et al. [5] who performed an initial screening of fifteen local plant species to explore their potential as a mosquito larvicidal agent against early fourth instars of dengue vector* Ae. aegypti *and reported the effectual larvicidal potential of hexane extracts of selected plant species resulting in 100% mortality at 1000 ppm. They observed that the extracts made from different parts of* A. aspera, C. occidentalis, L. camara, Ricinus communis, Trachyspermum ammi*, and* Zingiber officinalis *possessed significant larvicidal potential with LC_50_ values ranging from 30.00 ppm to 74.67 ppm. Likewise, Kumar et al. [16] when assaying different parts of a weed,* Parthenium hysterophorus*, extracted in different solvents against* Ae. aegypti* larvae found only hexane and petroleum ether extracts effective causing 100% mortality.

Likewise, evaluation of the larvicidal activity of extracts prepared from fourteen medicinal plants against* Ae. aegypti *revealed that eight out of fourteen plant species resulted in 100% larval mortality with their LC_50_ values ranging between 4.1 *μ*g/mL and 89.4 *μ*g/mL [31]. Similar studies performed by Sakthivadivel and Daniel [32] evidently proved the toxicity of six plant extracts,* Acacia nilotica* (leaf),* A. mexicana *(leaves and seeds),* Citrullus colocynthis* (leaf),* Jatropha curcas* (leaf), and* Withania somnifera* (leaf), resulting in an LC_50_ value of less than 100 ppm against 3rd instars of* Cx. quinquefasciatus*,* An. stephensi*, and* Ae. aegypti*. Comparable screening assays with a total of 94 extracts prepared from ten plant species belonging to eight families widely found in the Northeast of Brazil were performed by Oliveira et al. [33]. The assays were carried out with 250 *μ*g/mL extracts against fourth instars of* Ae. aegypti* which showed significant toxicity (>75%) of nineteen extracts from six plant species,* Coccoloba mollis, Guettarda grazielae, Merremia aegyptia, Rourea doniana, Spermacoce verticillata*, and* Triplaris americana*. Contrary results were presented by Yang et al. [34] who studied the cidal effects of polar methanol extracts, prepared from 28 medicinal plant species, against early 4th instars of* Ae. aegypti* and observed 100% larval mortality at 100 ppm methanol extracts of* Kaempferia galanga *rhizome.

Our results confirmed greater larvicidal potential of nonpolar hexane extracts of all the five plant species against* Ae. aegypti* than the polar ethanol extracts which led us to assess their effectiveness at various concentrations. Our studies are in conformity with that of Rahuman et al. [35] who obtained better potency of nonpolar extracts than the polar extracts when they assayed the ethyl acetate, butanol, and petroleum ether extracts of five species of Euphorbiaceae family,* Jatropha curcas, Pedilanthus tithymaloides, Phyllanthus amarus, Euphorbia hirta*, and* E. tirucalli *against the early fourth instars of* Ae. aegypti*.

In contrast, Kalimuthu et al. [36] established the highest larval mortality in* Ae. aegypti* when exposed to the ethanol leaf extract of* Cadaba indica lam* exhibiting an LC_50_ value of 143.75 ppm compared with that resulting on exposure to hexane, chloroform, and petroleum ether extracts. However, results comparable to present investigations were reported by Maheswaran et al. [37] who tested the crude extracts of* Leucas aspera *for their larvicidal activity against* Ae. aegypti *and* Cx. quinquefasciatus *and observed hexane extract to possess the highest larvicidal activity against the two vectors followed by that of chloroform and ethanol. Likewise, the studies reported by Warikoo and Kumar [17] showed hexane and petroleum ether extracts of* Argemone mexicana* as effective larvicides resulting in 80–100% mortality at 1,000 ppm when assayed against fourth instars of* Ae. aegypti.* Other extracts, benzene, acetone, and ethanol, however, were found to be comparatively ineffective.

Present investigations clearly revealed the efficacy of hexane extracts prepared from the leaves and stem of* A. aspera *against early IV instars of* Ae. aegypti* as compared to the other four extracts. Similar results have been reported by Bhattacharya and Chandra [38] who assayed different instars of* Cx. vishnui *with acetone leaf extracts of* A. aspera *and reported LC_50_ values ranging from 35.46 ppm to 63.39 ppm. Earlier reports have shown the larvicidal efficacy of the extracts prepared from the leaves, flowers, and the seeds of* A. aspera *against the fourth instars of* An. subpictus *and* Cx. tritaeniorhynchus *[19]. In contrast, Kumar et al. [5] found the higher larvicidal efficacy of hexane leaf extract of* L. camara* and* C. occidentalis *exhibiting a significant LC_50_ value of 30.71 ppm and 74.67 ppm as compared to that of* A. aspera*. Rajasekaran and Duraikannan [39] also reported higher toxicity of petroleum ether and aqueous extract of* L. camara* against 4th instars of* Ae. aegypti* resulting in 100% mortality as compared to the chloroform extract which could result in only 36.89% mortality after 24 hours. The petroleum ether and n-butanol extracts of* C. occidentalis *are also found to be more selective and biodegradable agents with appreciable larvicidal potential against filarial vector* Cx. quinquefasciatus* [23].

## 5. Conclusions

Our investigations have established the potential of hexane leaf extracts prepared from different plant species against early fourth instars of* Ae. aegypti* and their possible use in the development of larvicides for mosquito management. The variety of types and levels of active constituents in each kind of extract may be responsible for the variability in their potential against* Ae. aegypti*. However, the mechanism involved causing mortality of mosquito larvae is still unknown and needs to be studied further. Our investigations need further exploration to find out and identify the bioactive components involved and their mode of action.

## Figures and Tables

**Table 1:** Screening of the 1000 ppm hexane and ethanol extracts of five plant species for their larvicidal activity against early fourth instars of dengue vector *Aedes aegypti*.

Name of the plant species	Local name	Family	Part used	% mortality after 24 h	
				Hexane extract	Ethanolic extract
*Achyranthes aspera*	Prickly chaff flower	Amaranthaceae	Leaves	100.0 ± 0.0^*∗*^	11.0 ± 0.0
			Stems	100.0 ± 0.0	19.0 ± 0.33
*Cassia occidentalis*	Chakunda; Coffee Senna; Coffee Weed	Fabaceae	Leaves	100.0 ± 0.0	16.0 ± 0.0
			Stems	100.0 ± 0.0	10.0 ± 0.0
*Catharanthus roseus*	Periwinkle	Apocynaceae	Leaves	100.0 ± 0.0	50.0 ± 0.0
			Stems	100.0 ± 0.0	05.0 ± 0.0
*Lantana camara*	Spanish flag; Wild Sage; West Indian lantana	Verbenaceae	Leaves	100.0 ± 0.0	11.0 ± 0.0
			Stems	100.0 ± 0.0	05.0 ± 0.0
*Xanthium strumarium*	Common cocklebur; chotadhatura	Asteraceae	Leaves	100.0 ± 0.0	41.0 ± 4.33
			Stems	100.0 ± 0.0	47.0 ± 4.0

^*∗*^Mean ± SEM.

**Table 2:** Larvicidal potential of the leaf hexane extracts of the selected plant species against early fourth instars of dengue vector *Aedes aegypti*.

Name of the plant	LC_50_ (ppm)	95% fiducial limits	LC_90_ (ppm)	95% fiducial limits	Regression coefficient	SE	*χ* ^2^	DF
*Achyranthes aspera*	82.555	73.554–91.860	139.817	120.892–177.653	5.600	0.851	6.738	6
*Cassia occidentalis*	117.451	104.353–138.047	169.345	142.818–235.218	8.064	1.515	1.569	3
*Catharanthus roseus*	86.913	75.720–102.674	156.581	126.011–238.592	5.012	0.92	5.978	4
*Lantana camara*	118.499	105.520–145.823	159.985	134.032–242.868	9.83	2.236	2.36	2
*Xanthium strumarium*	586.185	518.761–649.958	851.821	754.752–1045.942	7.895	1.403	0.561	3

**Table 3:** Larvicidal potential of the stem hexane extracts of the selected plant species against early fourth instars of dengue vector *Aedes aegypti*.

Name of the plant	LC_50_ (ppm)	95% fiducial limits	LC_90_ (ppm)	95% fiducial limits	Regression coefficient	SE	*χ* ^2^	DF
*Achyranthes aspera*	68.133	42.340–92.723	115.075	86.689–440.534	5.630	0.901	15.910	5
*Cassia occidentalis*	149.698	135.101–166.024	206.337	182.760–257.751	9.195	1.724	0.433	3
*Catharanthus roseus*	108.245	82.320–196.106	184.239	128.299–895.320	5.548	0.980	7.609	4
*Lantana camara*	89.621	81.433–102.569	125.892	108.091–180.782	8.683	1.982	1.226	3
*Xanthium strumarium*	460.923	375.932–554.572	1074.014	839.391–1638.379	3.488	0.571	2.675	4
